# Modification and crosslinking strategies for hyaluronic acid‐based hydrogel biomaterials

**DOI:** 10.1002/SMMD.20230029

**Published:** 2023-10-30

**Authors:** Zhiqiang Luo, Yu Wang, Ye Xu, Jinglin Wang, Yunru Yu

**Affiliations:** ^1^ Department of Rheumatology and Immunology Nanjing Drum Tower Hospital School of Biological Science and Medical Engineering Southeast University Nanjing China; ^2^ Pharmaceutical Sciences Laboratory Åbo Akademi University Turku Finland

**Keywords:** chemical modification, covalent crosslinking, dynamic covalent crosslinking, hyaluronic acid, hydrogel, physical crosslinking

## Abstract

Hyaluronic acid (HA) is an attractive extracellular matrix‐derived polymer. The related HA‐based hydrogels are emerging to be the hotspots in the cutting edge of biomaterials. The continuous sights concentrate on exploring modification methods and crosslinking strategies to promote the advancement of HA‐based hydrogels with enhanced physical/chemical properties and enriched biological performance. Here, the advances on modification methods and crosslinking strategies for fabricating HA‐based hydrogels with diverse capacities are summarized. Firstly, the modification reactions that occur on the active hydroxyl, carboxyl and N‐acetyl groups of HA molecule are discussed. Next, the emphasis is put on various crosslinking strategies including physical crosslinking, covalent crosslinking and dynamic covalent crosslinking. Finally, we provide a general summary and give a critical viewpoint on the remaining challenges and the future development of HA‐based hydrogels. It is hoped that this review can provide new proposals for the specific design of functional hydrogel biomaterials.

## Graphical abstract

The advances on modification methods and crosslinking strategies for fabricating hyaluronic acid‐based hydrogels with diverse capacities are summarized. The emphasis is put on various crosslinking strategies including physical crosslinking, covalent crosslinking and dynamic covalent crosslinking. A general summary and a critical viewpoint are provided on the remaining challenges and the future development of hyaluronic acid‐based hydrogels.

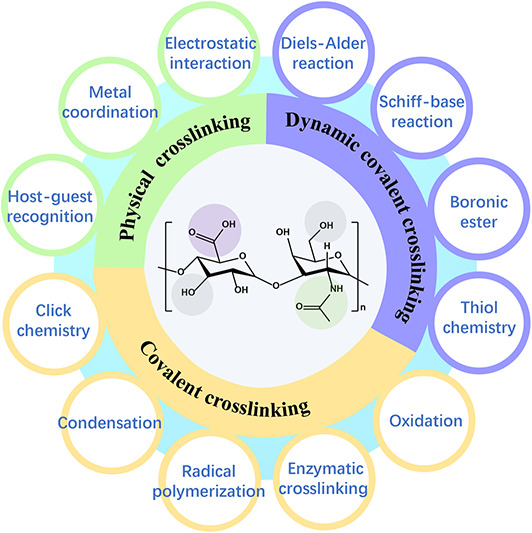

**Key points**

- The advances on modification methods and crosslinking strategies for fabricating HA‐based hydrogels are summarized.
- The emphasis is put on various crosslinking strategies including physical crosslinking, covalent crosslinking and dynamic covalent crosslinking.

## 1 INTRODUCTION

Hyaluronic acid (HA) is a naturally acidic mucopolysaccharide in extracellular matrix (ECM), consisting of a repeating unit of disaccharide, namely β‐1,4‐D‐glucuronic acid‐‐β‐1,3‐N‐acetyl‐D‐glucosamine.^1^ It exhibits endogenous curative effects to diverse diseases and has targeted features to cluster of differentiation‐44 (CD44) receptors.^2^, ^3^ Particularly, HA hydrogels have received extensive attention, which possess biodegradable three‐dimensional (3D) polymer networks resembling to ECM.^4^ They are regarded as promising biomaterials to construct artificial organs, promote injury repair and treat cancer.^5^ However, due to their sensitivity to hyaluronidase, the natural HA hydrogels are more vulnerable than synthetic polymers, limiting their biomedical applications. To resolve this issue, various modification strategies have been put forward, providing proposals for the fabrication of HA‐based hydrogels with enhanced properties and multifunction. Owing to the presence of linear molecular chain and the available functional groups including carboxyl, hydroxyl, and N‐acetyl on the HA chain, diverse kinds of modification reactions can be achieved.^6^ These reactions involve the introduction of functional groups into HA chain such as hydrazide, methacrylate, thiol, tyramine and catechol.^7^ These modified functional groups are not only beneficial for the therapeutic incorporation, but also facilitate the crosslinking of these polymers to enhance their biological activities.^8^

In addition, a number of crosslinking approaches are proposed, taking advantage of the added functional groups to facilitate the polymerization of HA‐based hydrogels in a high efficiency and low toxicity routine.^9^ The cross‐linking process may significantly influence the physicochemical properties of HA‐based hydrogels.^10^ Based on the specific polymer interactions, these crosslinking methods are normally distinguished as physical crosslinking, covalent crosslinking and dynamic covalent crosslinking (DCC).^11^ In general, physical crosslinking leads to a rapid polymerization process under relatively mild preparation conditions and is normally reversible.^12^ Chemical approaches can precisely control the crosslinking procedure to fabricate hydrogels with tailored flexibility and spatiotemporal precision.^13^ DCC forms adaptable and reversible polymer networks with tunable physical and chemical characteristics, imparting hydrogels with injectability in situ and stimuli responsiveness.^14^ These characteristics support the customed fabrication of HA‐based hydrogels to meet the various biomedical demands. Although great achievements have been made, there is a lack of systematic review to highlight the recent progress in terms of modification methods and crosslinking strategies of HA‐based hydrogels.

Herein, we will review the recent advances concerning HA‐based hydrogels. Firstly, the focus is concentrated on various chemical modifications to HA, mainly involving several functional groups including hydroxyl, carboxyl and N‐acetyl. Then, we systematically present crosslinking methods for the fabrication of HA‐based hydrogels, including physical crosslinking, covalent crosslinking and DCC. Ultimately, a summary and the future optimization directions are proposed, which will analyze and discuss current challenges and perspectives about the future HA‐based hydrogels.

## 2 CHEMICAL MODIFICATION

The hydrogels of natural HA are commonly crosslinked by condensation crosslinking of HA chains, which will be discussed in Section 3.2.1. However, they have the disadvantages of poor stability, weak mechanical strength, sensitivity to hyaluronidase and free radicals. To expand their applications in numerous biomedical fields, the chemical modification of the molecule chain is often needed before crosslinking.^15^, ^16^ Modifications on HA are easy‐going due to the linear nature and the available carboxyl, hydroxyl, and N‐acetyl groups on HA chain.^6^ Most of them occur on the active hydroxyl groups and carboxyl groups, including methacrylate modification, tyramine modification, thiol modification and hydrazide modification, etc.^7^ The primary reactions occurring on the carboxyl groups include the creation of ester and amide bonds in the presence of condensing agents while that on the hydroxyl groups include the formation of ester and ether bonds. The modified HA allows the hydrogel formation of polymer networks with significantly changed chemical/physical properties.^8^ These hydrogels may be imparted with increased enzymatic stability, altered viscoelastic behavior and tunable mechanical strength while the capacities of biodegradability and biocompatibility of natural HA may still be retained.^6^, ^8^

### 2.1 Modification of hydroxyl

**FIGURE 1:**
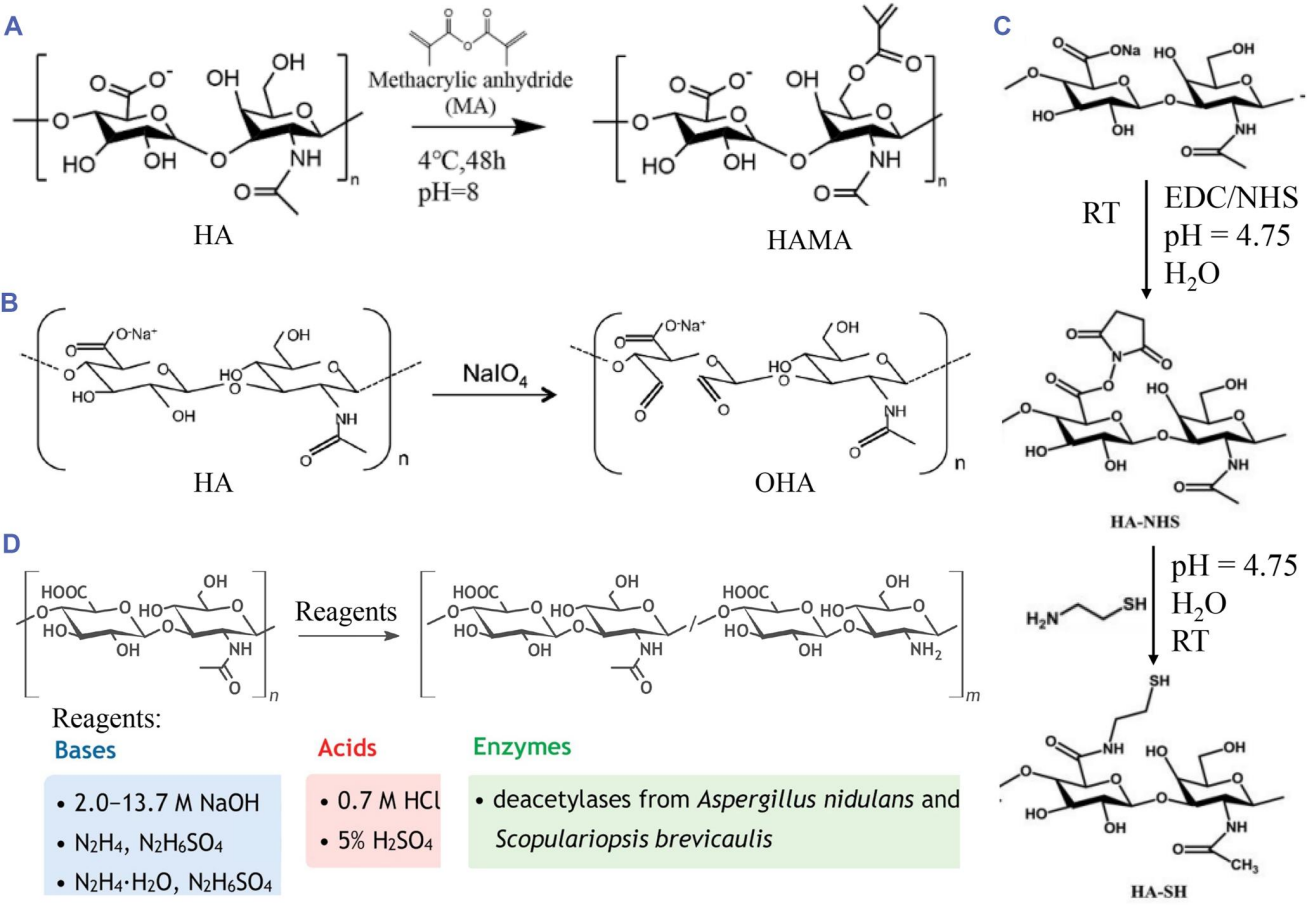
Chemical modifications of HA. (A) Esterification reaction on hydroxyl groups. Reproduced with permission.^17^ Copyright 2021, American Chemical Society. (B) Oxidation reaction on hydroxyl groups. Reproduced with permission.^18^ Copyright 2017, Elsevier. (C) Amidation rection on carboxyl groups by carbodiimide chemistry. Reproduced with permission.^19^ Copyright 2016, Elsevier. (D) Deacetylation of HA on the N‐acetyl group. Reproduced with permission.^20^ Copyright 2020, Elsevier.

The main modifications on hydroxyl groups include etherification, esterification, and oxidation reaction.^6^ Etherification is one of the universal methods to modify HA through the hydroxyl groups. Traditional crosslinkers like divinylsulfone (DVS) and glutaraldehyde (GTA) react with hydroxyl groups to form ether bonds. In basic solutions, epoxides can react with hydroxyl groups of HA to form ethers. Esterification can work between methacrylic anhydride and HA, generating methacrylated HA (HAMA), which could be used for further photo‐crosslinking (Figure 1A).^17^, ^21^ Besides, ester bonds are formed by the reactions of octenyl succinic anhydride with hydroxyl of HA under alkaline conditions, imparting HA with amphiphilic property.^22^ In contrast, an oxidation reaction can be performed through the oxidation of primary hydroxyl on HA by sodium periodate, opening the six‐membered ring and finally forming dialdehyde, namely oxidized HA (OHA) (Figure 1B).^7^, ^18^

### 2.2 Modification of carboxyl

The modification of carboxyl groups includes esterification and amidation. Esterification is usually induced by small molecule crosslinkers for condensation crosslinking, while amidation is often carried on the basis of carbodiimide chemistry. The formation of an amide bond occurs using two continuous processes: the carboxyl group is activated by an activator, forming an activated adder that is then attacked by amino groups. The activator is not introduced into the HA chain, which facilitates bonding within the structure. The previous studies have reported many activators to condense amino and carboxyl groups including carbodiimides, carbonyldiimidazole, etc.^6^ Due to good water solubility and biocompatibility, the combination of 1‐ethyl‐3‐[3‐(dimethylamino)‐propyl]‐carbodiimide (EDC) and N‐hydroxysuccinimide (NHS) is the preferred activator (Figure 1C).^19^ This reaction is typically utilized to conjugate HA with other polymers, therapeutic, or detect probes.^7^ For instance, hydrazide‐modified HA was obtained when dihydrazide adipate was coupled to the carboxyl of HA activated by EDC/NHS. It was able to form hydrazone bonds with aldehydes as well as ketones, and formed amide bonds with the carboxyl, which was pH‐responsive.^7^, ^23^, ^24^ Similarly, tyramine modification and thiol modification of HA can occur in the same way. The tyramine‐HA (HA‐Tyr) allows self‐crosslinking under catalyzation of horseradish peroxidase (HRP). The thoil‐modified HA (HA‐SH) can undergo oxidation, disulfide exchange, radical polymerization and thiol‐Michael addition reactions for crosslinking.^25^, ^26^

### 2.3 Modification of N‐acetyl

The main modification reactions on the N‐acetyl group are deacetylation and amidation.^6^ N‐deacetylated HA is commonly obtained by hydrazinolysis between HA and hydrazine sulfate.^27^ Treatments with bases, acids and deacetylases have also been reported (Figure 1D).^20^ However, deacetylation may cause chain fragmentation of HA molecule. Deacetylation of N‐acetyl group produces an amino group which can be used as a reaction site for chemical conjugation of the acid derivatives via amide formation.^7^ The amine groups of N‐deacetylated HA can also be reacted with hydroxyl of HA to produce auto‐crosslinked hydrogels.^28^

## 3 CROSSLINKING STRATEGIES

The main crosslinking strategies involve adding small cross‐linkers, using activated HA that is modified with functional groups and combined ways.^9^ Based on forming covalent or non‐covalent bonds, polymers can be crosslinked into physical and chemical hydrogels. The crosslinking methods include physical crosslinking, covalent crosslinking and DCC. The actions of cross‐linking can alter the performances of HA polymers, such as increasing molecular chain length, viscoelasticity and mechanical strength while weakening their relative water solubility.^10^ The resultant HA‐based hydrogels form 3D polymeric networks with tailored mechanical strength, stability and degradation rate. Meanwhile, the obtained hydrogels are still able to possess naturally biological functions, versatility of HA itself and inherent biocompatibility. These features make them widely applied in tissue engineering, wound administration, target drug delivery and others.^29^

### 3.1 Physical crosslinking

The physical crosslinking process normally depends on the inherent features of the HA and the introduced groups, which does not form new covalent bonds.^30^ Physical interactions, including electrostatic interactions, chain entanglement, hydrophobic self‐assembly, hydrophobic/hydrophilic interactions, and hydrogen bonds, have been developed as common crosslinking strategies for the preparation of HA‐based hydrogels.^31^ Physical crosslinking normally leads to a rapid polymerization behavior under relatively mild conditions and needs no toxic crosslinkers or catalysts, decreasing potential cytotoxicity.^12^, ^25^ Since physical interactions are reversible, they have the potential to design injectable hydrogels with the capacities of shear‐thinning and self‐healing for extrusion bioprinting and injection.^32^, ^33^ Besides, they are sensitive to external stimuli such as temperature, enzymes, pH, and free radical.^25^ These features make them suitable to deliver drugs and encapsulate living cells. However, the gelling process is hard to be accurately controlled and the mechanical and chemical stabilities of the resultant hydrogels are poor. They may lose their structural integrity with the change of environmental parameters.^25^

**FIGURE 2:**
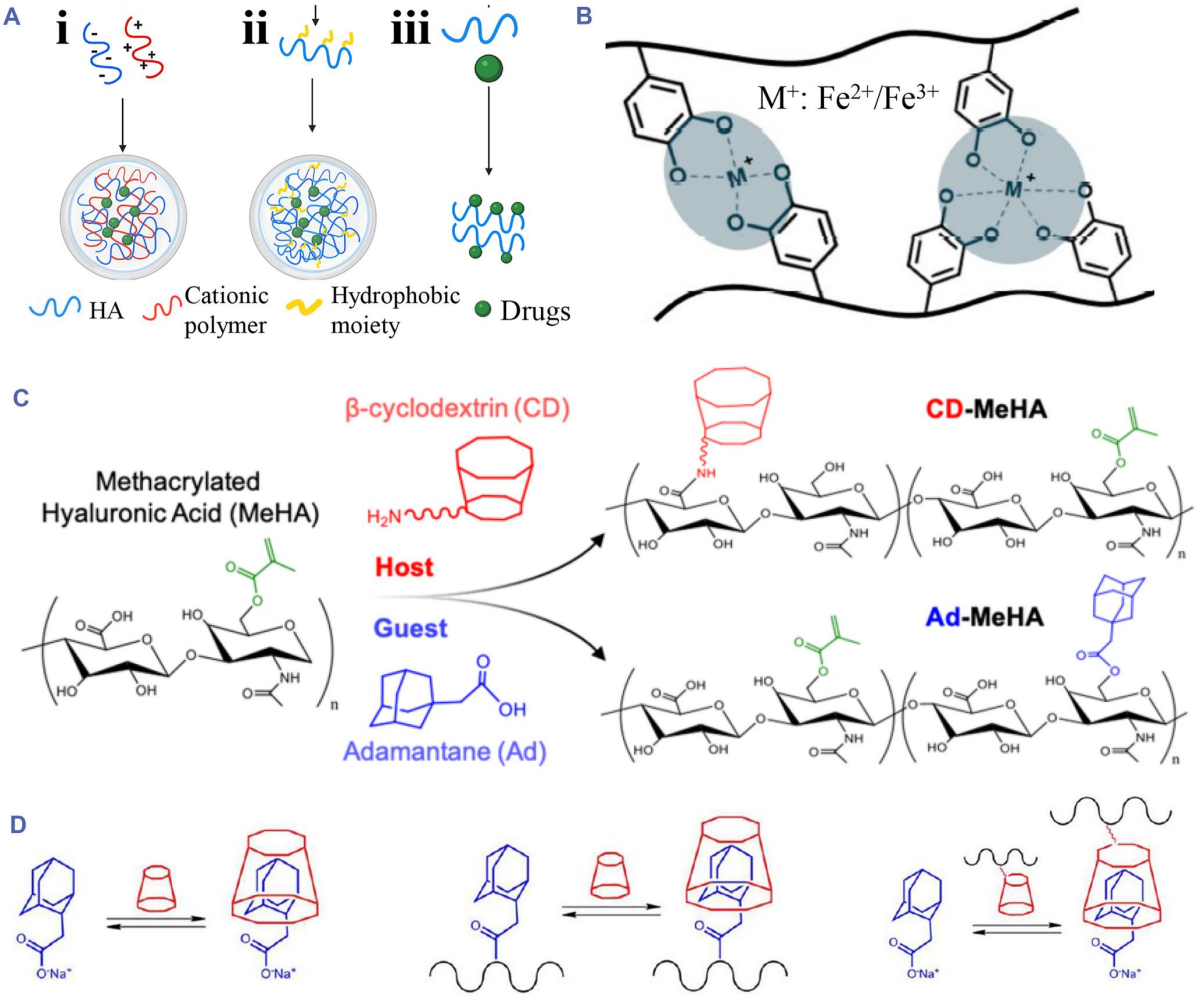
Physical crosslinking methods. (A) Electrostatic interaction (i), hydrophobic self‐assembly (ii), and hydrophobic/hydrophilic interaction (iii). Reproduced under terms of the CC‐BY license.^34^ Copyright 2022, The Authors, published by John Wiley and Sons. (B) Metal coordination of HA‐CA. Reproduced under terms of the CC‐BY license.^35^ Copyright 2021, The Authors, published by MDPI. (C) The fabrication and chemical structure of HA‐β‐cyclodextrin and HA‐adamantane. Reproduced with permission.^36^ Copyright 2021, American Chemical Society. (D) The crosslinking scheme for host‐guest recognition of adamantane and β‐cyclodextrin. Reproduced with permission.^37^ Copyright 2013, American Chemical Society.

Physical crosslinking methods mainly include electrostatic interaction, metal coordination and host‐guest recognition.^29^ Owing to the anionic nature of HA, specific positive ion and cationic polymers can be used to fabricate hydrogel via electrostatic interactions.^34^ Blending HA with cationic polymers like chitosan (CS) is a common method for preparing hybrid HA hydrogel (Figure 2Ai).^25^, ^38^ The formation of inhomogeneous large aggregates is a major challenge in this process due to the violent electrostatic interaction of oppositely charged polyelectrolytes.^30^ In another interesting method, hydrophobic moieties like cholesterol are coupled into hydrophilic HA to manifest amphiphilicity, allowing the self‐assembly of macromers into hydrogels (Figure 2Aii).^39^ Recent research reported that mixing HA with specific hydrophobic molecule (e.g. hydrophobic drugs) could also form hydrogels (Figure 2Aiii).^34^ In addition, catechol‐modified HA (HA‐CA) is reported to be crosslinked through metal coordination between iron ions (Fe^2+^ and Fe^3+^) and catechol groups (Figure 2B).^4^, ^35^

Guest‐host interaction, also named inclusion complexation, is a promising method to fabricate the physically crosslinked hydrogels. Based on the specific recognition effect of guest monomers on the host monomers, the crosslinking is induced by the self‐assembly behavior of guest/host monomers‐modified polymer molecules.^40^, ^41^ Most commonly, the host monomer owns a cavity accommodated by the guest monomer.^8^ The binding capacity among these molecules is decided by complementary structural features and hydrophobic interactions.^42^ On this coordination of guest‐host interaction, cyclodextrin is the most reported host monomer, while the guest monomer is adamantane.^43^ HA could be functionalized with both adamantanes via controlled esterification and β‐cyclodextrins through amidation (Figure 2C).^36^ They could form a HA hydrogel or other hybrid hydrogels via noncovalent guest‐host interactions (Figure 2D).^37^ These systems result in the self‐healing hydrogels due to the dynamically controllable hydrogel properties under physiological conditions.^25^ Analogously, azobenzene‐functionalized and β‐cyclodextrin‐modified HA produced a supramolecular hydrogel with shear‐thinning property at physiological conditions, whose capacities can be dynamically tailored.^44^ This mechanism can be utilized to deliver cargoes to the target site with high‐retention during injection and allows the integration of another covalent crosslinking process for subsequent stabilization of network.^45^ In general, these advantages of the guest‐host interaction impart resultant HA‐based hydrogels with self‐healing properties after extrusion, enabling their utilization as injectable matrix and bio‐inks for spatially controlled bioprinting applications and allowing the encapsulation of therapeutic molecules and cells.^8^

### 3.2 Covalent crosslinking

Chemical crosslinking strategies mainly include small molecular crosslinkers‐induced condensation crosslinking, self‐crosslinking of macromolecules, enzymatic crosslinking, photo‐crosslinking, etc.^29^ Chemical approaches can precisely control the crosslinking process and hydrogel structure.^30^ The resultant HA‐based hydrogels are more elastic than physical gels due to the formation of stable covalent bonds, while physical gels are normally more viscous.^46^ Compared with physical crosslinking, the gel networks of the obtained hydrogels have substantially improved flexibility and spatiotemporal precision.^13^

#### 3.2.1 Condensation reactions

**FIGURE 3:**
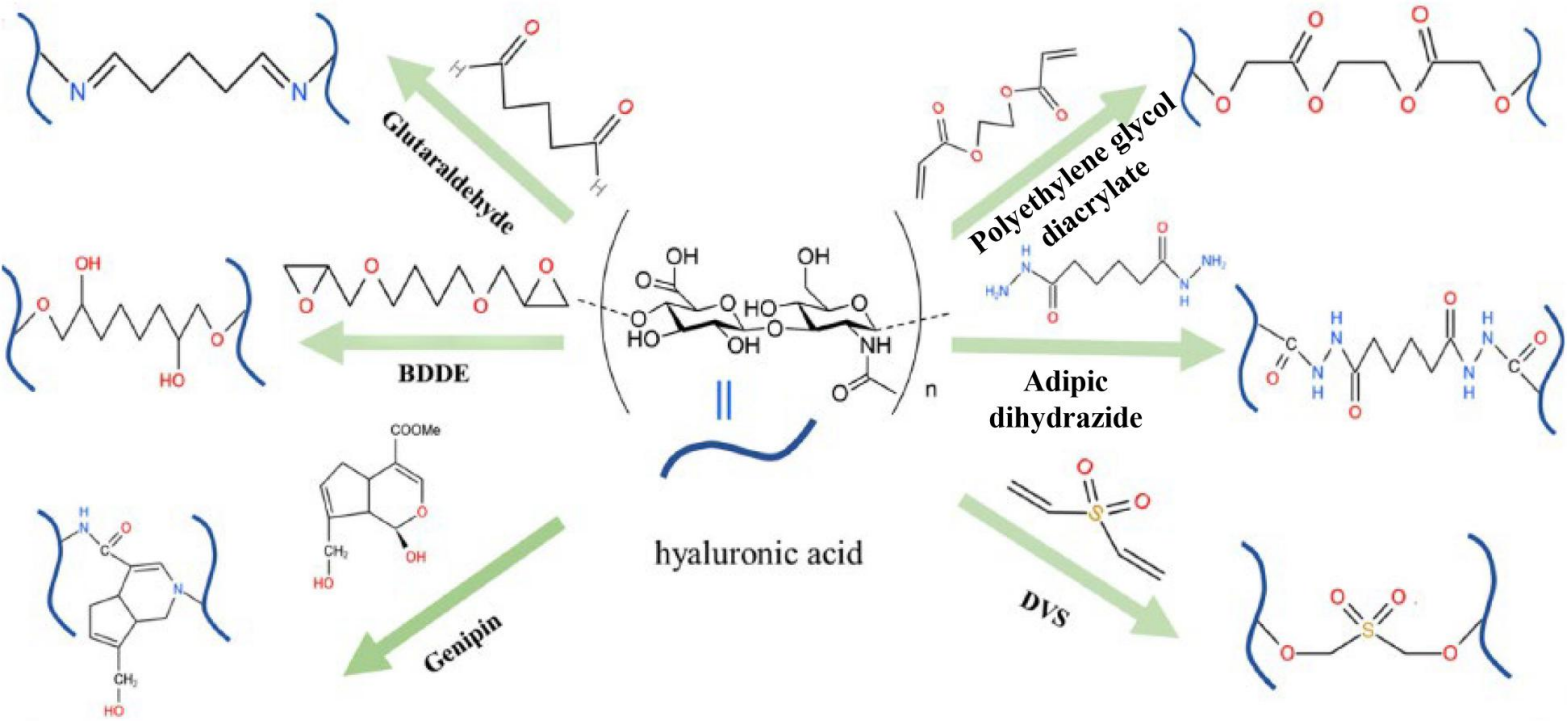
The commonly used small molecular crosslinkers for condensation crosslinking. Reproduced with permission.^29^ Copyright 2023, Elsevier.

Amidation on the carboxyl and etherification on hydroxyl group are the most common condensation crosslinking methods used for HA hydrogel fabrication, presented in Figure 3.^29^, ^47^ Briefly, the versatile crosslinkers for hydroxyl crosslinking are epoxides, aldehydes, DVS, GTA, etc. 1,4‐butanediol diglycidyl ether (BDDE) is considered as a universal epoxide crosslinker.^48^ It opens the ring of epoxy groups under alkaline condition and reacts with hydroxyl groups of HA to form a stable covalent ether for linkage.^49^ HA hydrogels prepared using this method are mostly used in the field of cosmetic filling. DVS‐induced crosslinking forms covalent ether bonds as well under alkaline conditions at room temperature.^50^ The obtained hydrogels are resistant to enzymatic degradation and biocompatible.^25^, ^51^ GTA reacts with hydroxyls under acidic conditions to form ether bonds, but this cross‐linking reaction is not as stable as that by DVS.^52^ The final product needs to be purified to avoid its toxicity.^6^ For carboxyl crosslinking, it is induced by EDC, hydrazine, and disulfide.^9^, ^53^ The biocompatibility of EDC crosslinked HA hydrogel is better than that of GTA.^54^

#### 3.2.2 Radical polymerization

**FIGURE 4:**
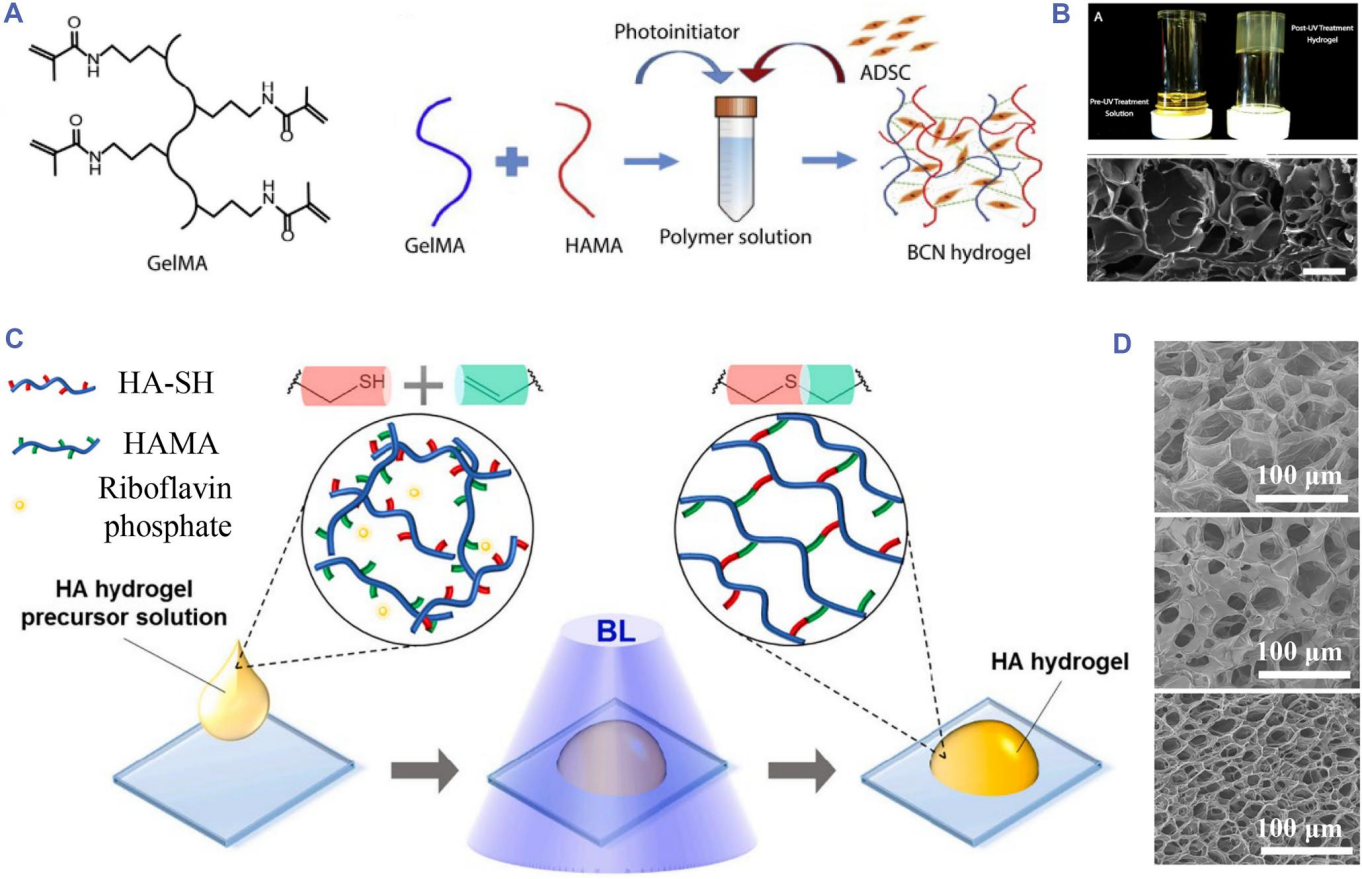
Radical polymerization for covalent crosslinking. (A) The chemical structure of GelMa and the principle diagram of photopolymerization between GelMa and HAMA. (B) The optical image and scanning electron microscope (SEM) image of GelMa‐HAMA hydrogel. (A,B) Reproduced with permission.^57^ Copyright 2017, Elsevier. (C) The principle diagram of photoinitiated polymerization between HA‐SH and HAMA. (D) The SEM images of hydrogels fabricated with varied ratios of HA‐SH and HAMA. (C,D) Reproduced with permission.^58^ Copyright 2021, Elsevier.

Radical polymerization is initiated by free radicals from the decomposition of the initiator under light, heat, and oxidative reactions, resulting in polymerization of monomers with specific double‐bond and other functional groups.^8^, ^29^ Using this strategy to prepare chemically crosslinked hydrogels has many advantages like good reaction kinetics, strong and tunable mechanical properties, ease of polymerization in situ and mild conditions for drugs loading.^2^, ^55^, ^56^ Photoinitiated polymerization is one of the most useful methods for fabrication of HA‐based hydrogels.^25^ Under the irradiation of visible or ultraviolet light, the photoinitiator generates free radicals to cause the cross‐linking reaction of polymers. Modified HA polymers with alkene, alkyne groups and thiol groups are mainly prepared into hydrogels through photoinitiation. HAMA and HA‐SH are the typical cases. The HAMA molecule is reported to form hydrogels by itself or other polymers with olefin bonds. For instance, methacrylate gelatin (GelMa) was utilized to fabricate GelMa‐HAMA hydrogel with porous structure, which could be crosslinked in situ under ultraviolet irradiation (Figure 4A,B).^57^

HA‐SA, obtained through HA thiolation, is a kind of HA derivative with free thiol groups. HA thiolation is normally achieved through coupling ligands with free thiol groups into either the carboxyl or hydroxyl groups on HA molecule.^25^ It can be crosslinked through photoinitiated polymerization as well. The thiol group of HA‐SH can undergo thiol‐ene reactions and thiol‐yne reactions to realize polymerization, mainly triggered by free radical from photoinitiators.^26^, ^30^ The mechanical properties of the resultant hydrogels are affected by various factor variations (e.g. chemical properties of initiators, concentration of initiators functionalities of monomers, temperature, light intensity, molecular weight, and solvents).^59^ For instance, HA‐SA and HAMA were used to prepare injectable hydrogels under the condition of blue light exposure (Figure 4C). The resultant hydrogel had a porous structure with interconnected pores, and the pore sizes were tunable by changing the ratios of HA‐SH and HAMA (Figure 4D).^58^ One of the limitations of such HA‐SH is the potential interaction between the free thiols in the system and the encapsulated proteins when it was developed to deliver therapeutic proteins, which may change the final function of loaded cargoes in vivo.^46^

In general, the fabrication of HA‐based hydrogels through radical polymerization is highly efficient and causes few side reactions. By accurately controlling crosslinking factors like the molecular weight of HA, monomer concentration, degree of modification, photoinitiator concentration, laser intensity and exposure time, the mechanical properties and biological functions of hydrogels are tunable.^25^, ^60^, ^61^, ^62^ The problems are that some photoinitiators themselves may be toxic and often produce extra free radicals in vivo, which lead to harmful side effects like local inflammation and unexpected side reactions.^29^ Fortunately, direct encapsulation of bioactive proteins, DNA and even cells is possible and promising once the initiation conditions are mild enough (e.g. biosafe photoinitiator, exact radical concentrations, suitable light intensities, etc.).^9^ The key issue is to compromise hydrogel properties and biocompatibility.

#### 3.2.3 Click chemistry

**FIGURE 5:**
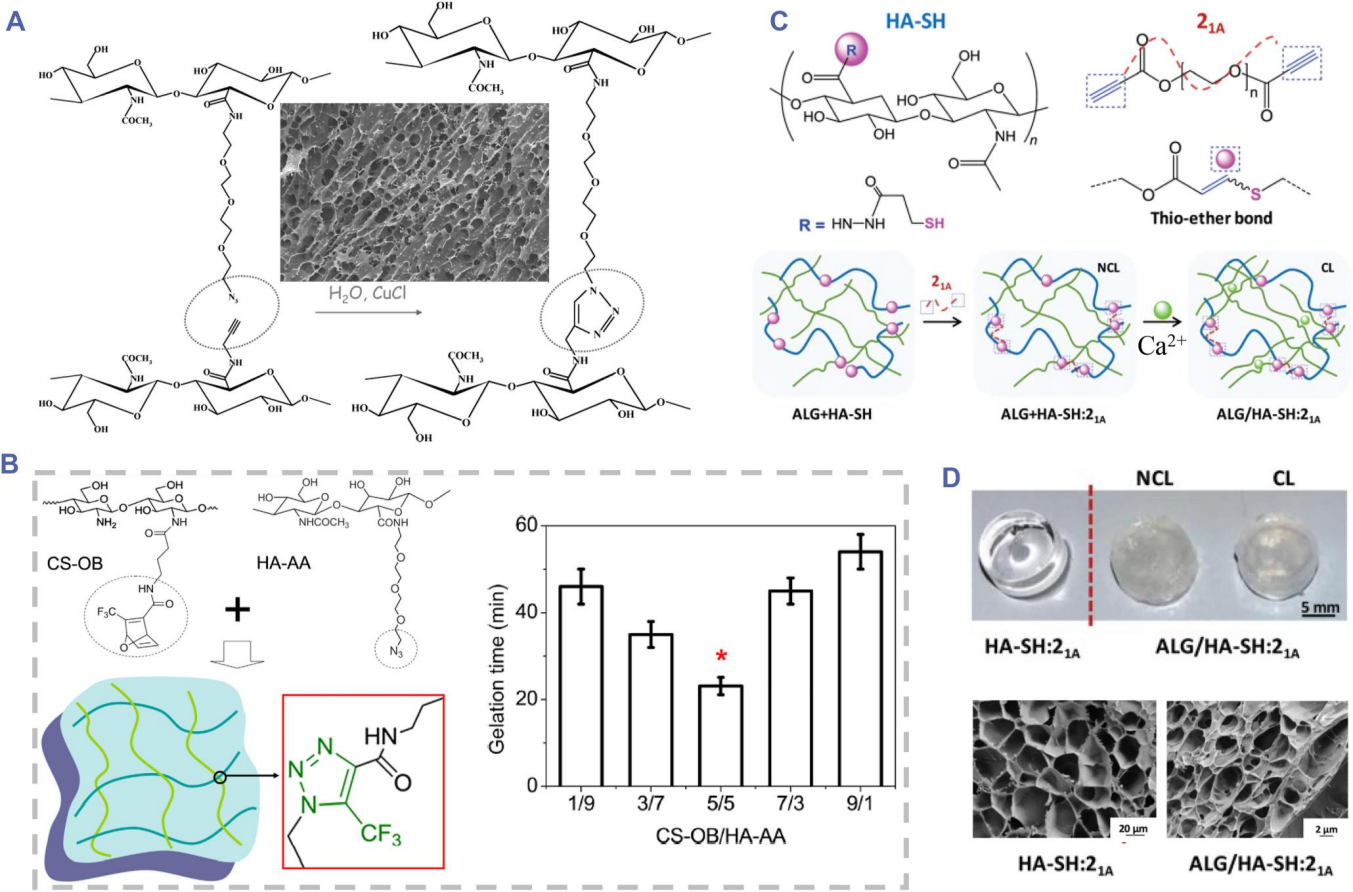
Click chemistry for covalent crosslinking. (A) The principle diagram of azide‐alkyne click reaction and the SEM image of resultant HA‐based hydrogel. Reproduced with permission.^65^ Copyright 2007, American Chemical Society. (B) The principle diagram of metal‐free click chemistry and the gelation time of the resultant HA‐based hydrogel fabricated with different volume ratios of HA‐AA and CS‐OB. Reproduced with permission.^68^ Copyright 2015, Elsevier. (C) The principle diagram of the thiol‐yne click reaction. (D) The optical and SEM images of the resultant HA‐based hydrogels added with and without alginate. (C,D) Reproduced under terms of the CC‐BY license.^69^ Copyright 2020, The Authors, published by Royal Society of Chemistry.

Click chemistry involves a series of chemical reactions that efficiently generate products with limited and easily removed byproducts, which occur under mild conditions due to the high thermodynamic driving force.^25^, ^63^ Azide‐alkyne cycloaddition and Diels‐Alder reactions are two kinds of click reactions used to HA‐based hydrogels.^6^ Diels‐Alder reaction can efficiently proceed under physiological conditions,^64^ discussed in Section 3.3.1 in detail. The Huisgen‐type cycloaddition reaction is a copper‐catalyzed azide‐alkyne reaction to produce triazoles cycloaddition, which has been used for crosslinking of modified HA.^65^ Based on carbodiimide chemistry, the carboxyl group on HA chain can be conjugated with either propargyl amides or 11‐azido‐triethyleneglycol amides. The click reaction occurs through a terminal alkyne and 1,3‐dipolar cycloaddition of azides.^66^ The selective catalyst Cu (I), commonly needs to be added to catalyze this reaction.^67^ For instance, propargylamine‐HA and 11‐azido‐3,6,9‐trioxaundecan‐1‐amine modified‐HA (HA‐AA) were synthesized and fastly crosslinked into gels (Figure 5A).^65^ Although the hydrogel was formed at room temperature, the toxicity of the used metal catalyst limited its clinically biomedical applications.^8^, ^70^ To overcome this challenge, click reaction of strain‐promoted azide‐alkyne cycloaddition obtained more and more attention because no catalyst is needed.^26^ Fan et al. reported that oxanorbonadiene‐modified CS (CS‐OB) and HA‐AA could be crosslinked in situ to form biocompatible hydrogel. The gel time was significantly influenced by the mixing ratios of CS‐OB/HA‐AA (Figure 5B).^68^ Fu et al. used cyclooctyne groups‐modified HA to react with azide‐polyethylene glycol (PEG) at physiological conditions, forming hydrogel with low toxicity and good biocompatibility.^71^

Nucleophilic thiol‐yne addition reaction is another kind of click reaction to prepare hydrogel with high cytocompatibility. This reaction is rapid and efficient, which is highly suitable for hydrogel synthesis.^72^ For example, the reaction between HA‐SH and PEG‐yne derivatives was used to prepare HA‐based hydrogels within a few synthetic steps (Figure 5C).^69^ Furthermore, alginate was added to strengthen the mechanical property of the resultant hydrogel, forming the second flexible network in the presence of calcium ions (Figure 5D). These HA‐SH hydrogels had great toxicological properties and improved stability, which were suitable to serve as drug delivery platforms toward target sites.^8^ These results support the promising potential of these approaches for preparing clickable, biocompatible, and more complex HA‐based hydrogels for clinical applications.

#### 3.2.4 Enzymatic crosslinking

**FIGURE 6:**
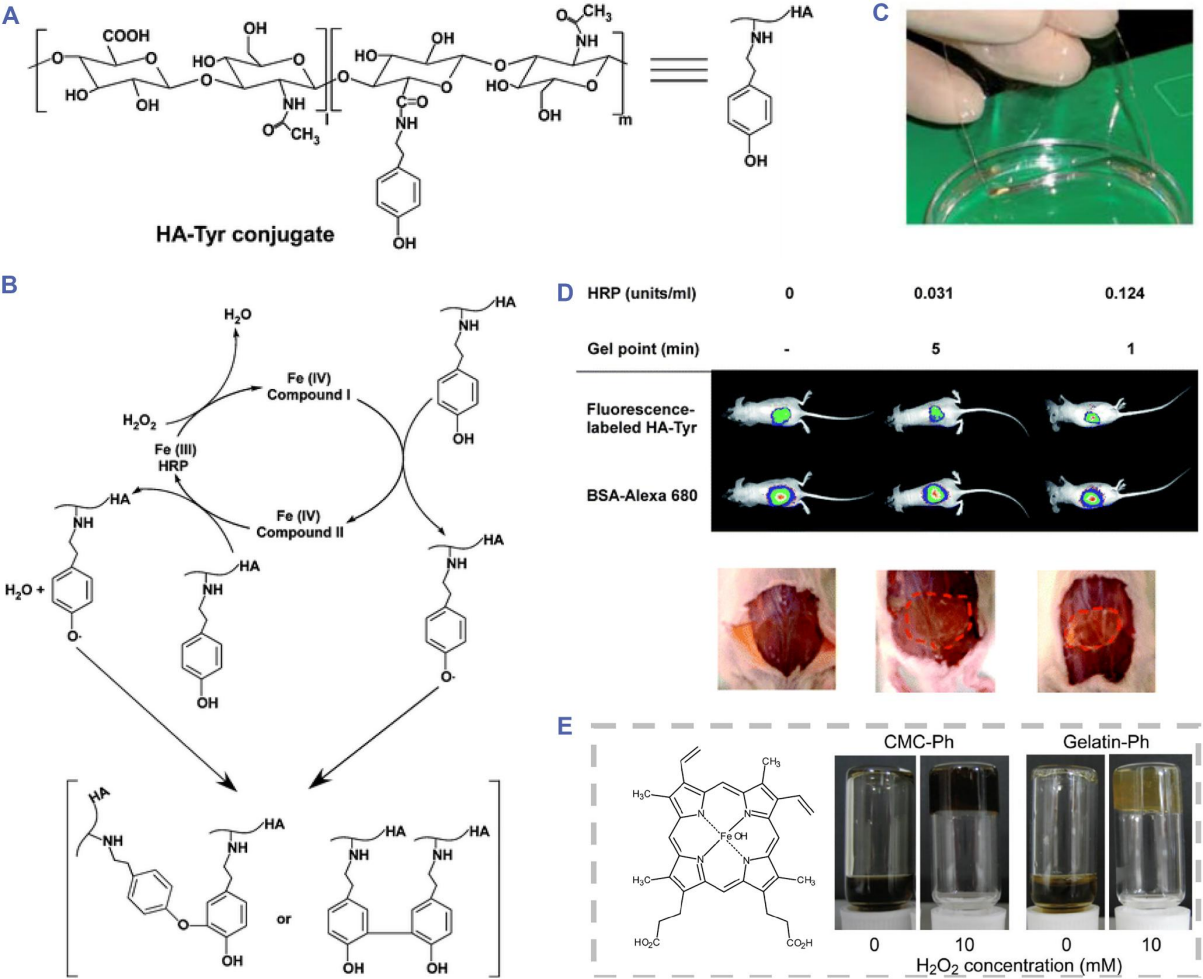
Enzymatic catalytic reactions for covalent crosslinking. (A) The chemical structure of HA‐Tyr. (B) The principle diagram of the reaction catalyzed by horseradish peroxidase. (C) The optical image of hydrogel in vitro. (D) The crosslinking performance of HA‐Tyr in vivo. (A–D) Reproduced with permission.^73^ Copyright 2010, Royal Society of Chemistry. (E) Chemical structure of hematin and its catalytic performance for crosslinking. Reproduced with permission.^74^ Copyright 2010, American Chemical Society.

Under the catalysis of HRP, tyramine can be enzymatically crosslinked due to the oxidation of the phenol group, producing free radicals. And in the presence of free radicals, carbon‐carbon and carbon‐oxygen coupling occur on the aromatic rings to result in crosslinking (Figure 6A,B).^75^ This reaction was used to crosslink HA‐Tyr together with hydrogen peroxide (H_2_O_2_) in vitro under the catalysis of HRP (Figure 6C).^76^ This system was developed to fabricate injectable hydrogels, which could be crosslinked under endogenous HRP (Figure 6D).^73^ Other mild catalysts like hematin have also been investigated to replace HRP to improve these crosslinking conditions (Figure 6E).^74^ Owing to mild reaction conditions, rapid and controlled gelation process,^73^ and controllable mechanical properties,^76^ enzymatic crosslinking is a promising strategy to fabricate HA‐based hydrogel.^77^ The desirable advantages of these gels make them suitable to be applied in multiple applications, including injectable matric platforms for drug and bioactive protein delivery, neural development, tissue engineering, etc.^25^, ^78^

#### 3.2.5 Oxidation

**FIGURE 7:**
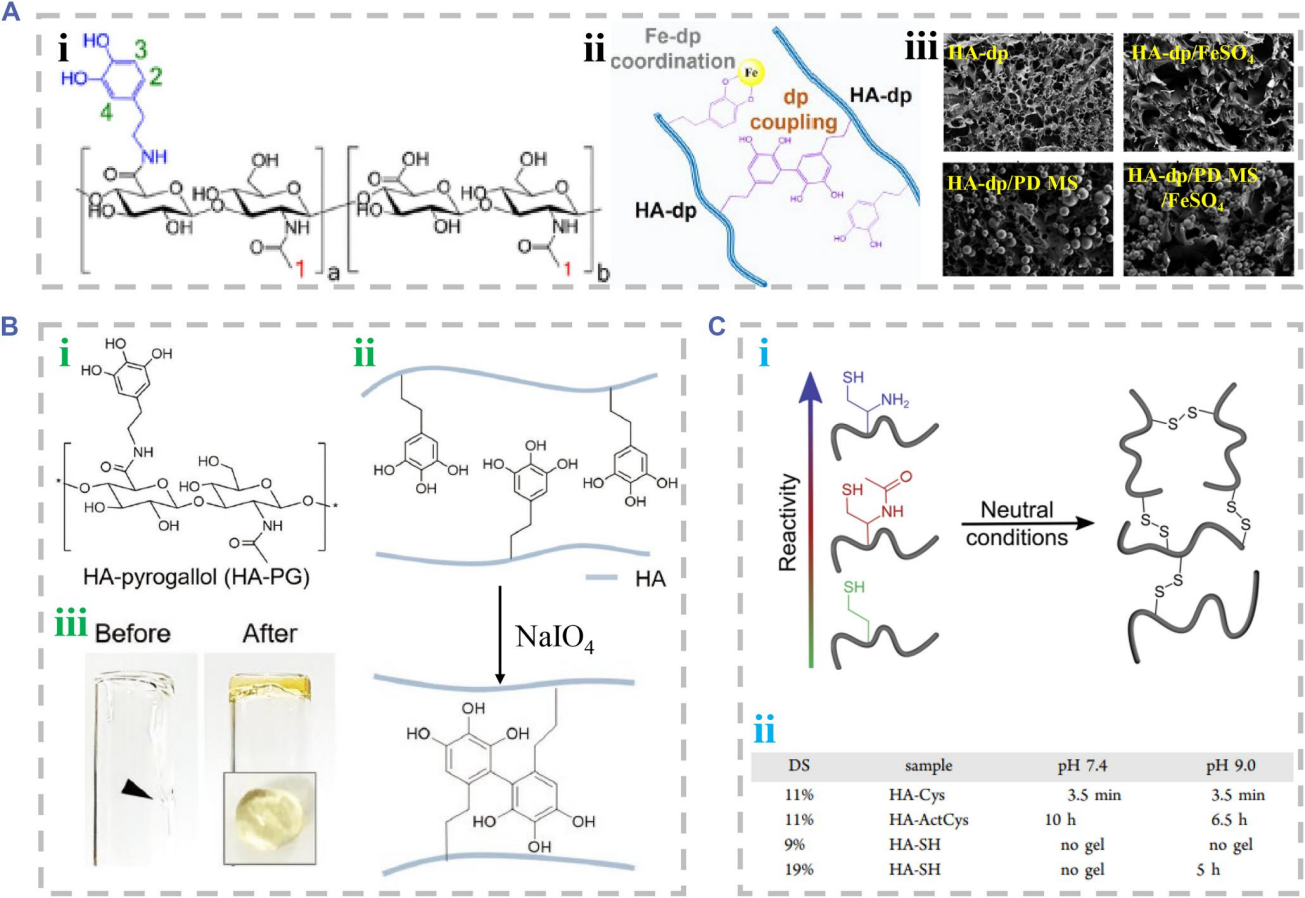
Oxidation reactions for covalent crosslinking. (A) The oxidation of HA‐CA. (i) The chemical structure of HA‐CA. (ii) The principle diagram of oxidation. (iii) The SEM images of HA‐CA hydrogel crosslinked using different methods. Reproduced with permission.^79^ Copyright 2020, Elsevier. (B) The oxidation of HA‐pyrogallol. (i) The chemical structure of HA‐pyrogallol. (ii) The principle diagram of oxidation. (iii) The optical images of HA‐pyrogallol hydrogel. Reproduced with permission.^80^ Copyright 2018, John Wiley and Sons. (C) The oxidation of HA‐SH. (i) The principle diagram of oxidation. (ii) The crosslinking performance of HA derivatives with different thiol groups and degree of substitution (DS). Reproduced with permission.^81^ Copyright 2019, American Chemical Society.

Catechol groups of HA‐CA participate in not only metal coordination with iron ions but also the oxidation reaction triggered by the oxidant.^4^ Oxidation reaction causes covalent catechol‐catechol coupling, which leads to polymerization of the polymers into stable hydrogels (Figure 7A).^79^, ^82^ Additionally, pyrogallol‐conjugated HA can be self‐crosslinked into HA‐pyrogallol hydrogel through the autoxidation of pyrogallol groups even without any chemical additives.^83^ Increase in pH value and addition of oxidant would trigger the polymerization of HA‐pyrogallol, which involves dual crosslinking of gallol groups like bicyclic gallol‐gallol adducts and biphenolic. Partial intermediates of the oxidation reaction of gallol groups such as purpurogallin‐quinone can react with thiol and amine groups of protein molecules on tissue surfaces via Schiff‐base reaction, which enhanced its tissue adhesiveness (Figure 7B).^80^ Additionally, an oxidation reaction may occur at the thiol groups of HA‐SH. The reaction rates are largely decided by the deprotonation degree of thiol groups under reaction conditions and the pKa of thiol groups.^19^ The formed oxidative disulfide bonds link HA‐SH polymers, obtaining disulfide hydrogels, which are influenced by the parameters of oxidant species radical initiators, the reaction pH value, and thiol deprotonation.^30^ Oxidants are the most important factor to precisely control the formation rate of disulfide. Introduction of the thiol with specific electron‐withdrawing group (EWG), like cysteine (Cys) and N‐acetyl‐L‐cysteine (ArtCys), may decrease its pKa, enhancing its reactivity (Figure 7C).^81^

### 3.3 DCC

DCC is the intermediary crosslinking method between physical and covalent crosslinking, which can be utilized to fabricate viscoelastic biomaterials with stress relaxation and deformation under stress.^11^ The bond energy of the formed dynamic covalent bonds is lower than that of the normal covalent bonds. These hydrogels with tunable physical and chemical properties possess reversible and adaptable polymer networks, commonly described as “dynamic hydrogels.”^84^ The network structure of the dynamic hydrogel system can be broken and healed because of the reversibility of “forming and breaking” state, performing shear‐thinning properties, stress relaxation, self‐healing capacity, and shape adaptability.^14^ Thus, structural changes of these hydrogels can occur along with the received external stimuli such as pH value, temperature, light irradiation, and induction of biomolecules or ligands.^85^, ^86^, ^87^ In a word, dynamic covalent crosslinked hydrogels may perform stimuli responsiveness and injectability in situ.^26^ These features allow the hydrogels to deform under the applied forces and regain their macroscopic characteristics when the applied force is removed, making them possible to be injected and printed.^46^ The mostly reported DCC methods, especially those with high reaction efficiency under mild conditions, will be summarized below.

#### 3.3.1 Diels‐Alder reaction

**FIGURE 8:**
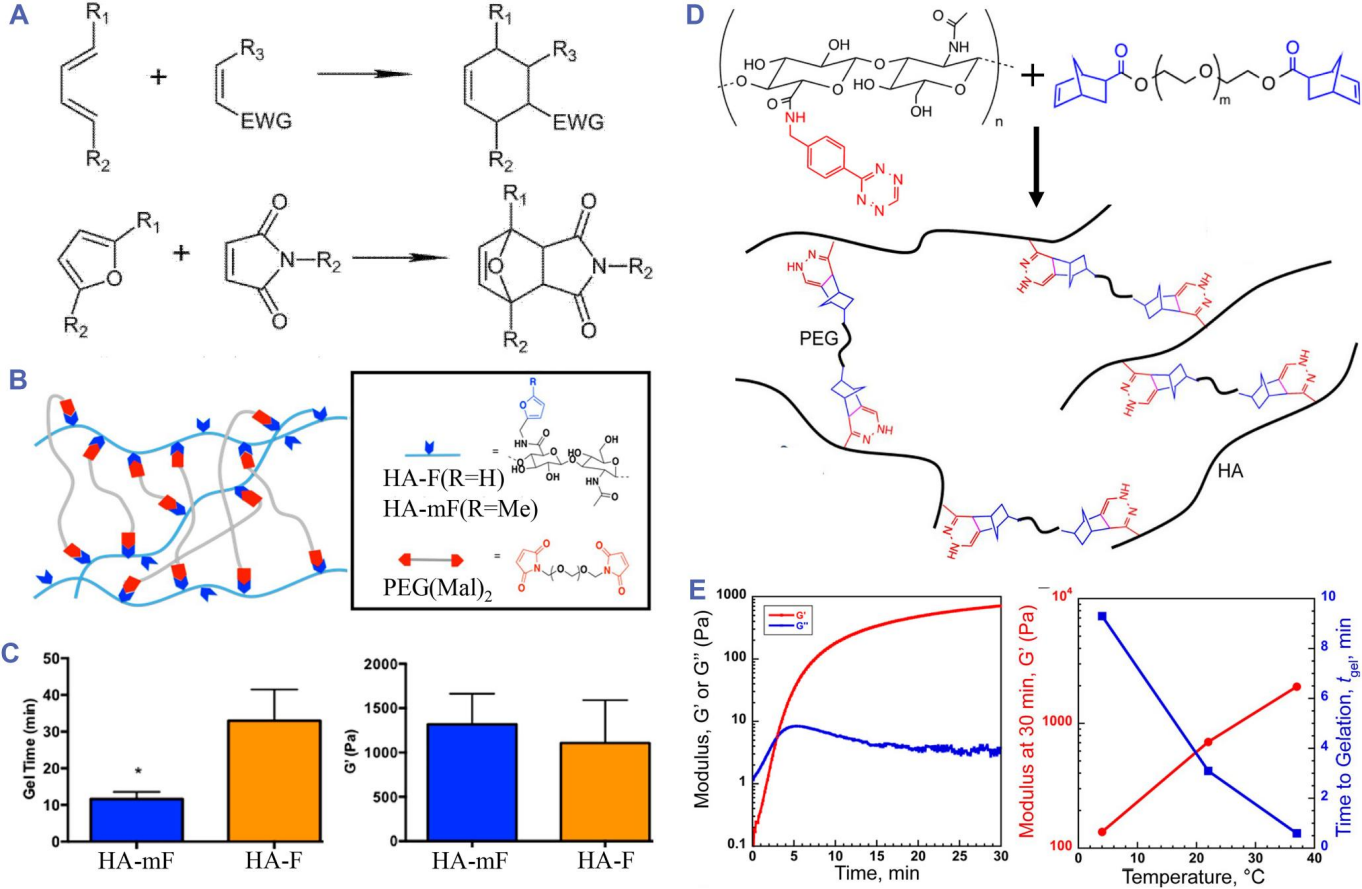
Diels‐Alder reactions for dynamic covalent crosslinking. (A) Chemical reactions based on the Diels‐Alder reaction. Reproduced under terms of the CC‐BY license.^26^ Copyright 2021, The Authors, published by MDPI. (B) Crosslinking diagram of HA‐F/HA‐mF and maleimide‐PEG. Reproduced with permission. (C) The gel time and modulus of HA‐F and HA‐mF. (B,C) Reproduced with permission.^88^ Copyright 2018, American Chemical Society. (D) The principle diagram of the inverse electron demand Diels‐Alder reaction. (E) The crosslinking performance of HA‐PEG. (D,E) Reproduced with permission.^89^ Copyright 2017, American Chemical Society.

This click reaction is a cycloaddition reaction with high selectivity between a diene and a dienophile, forming carbon‐carbon bonds to link molecules in the absence of a catalyst (Figure 8A).^90^ It can exhibit a dynamic temperature behavior under physiological conditions, suitable for several bioconjugation applications.^26^, ^90^ Furthermore, these Diels‐Alder reactions are reversible and negligibly sensitive to the existence of water, benefiting hydrogel preparation.^91^ Common Diels‐Alder reactions for HA crosslinking involve reactions between maleimide (dienophile) and furan (diene). Both furan‐functionalized PEG/maleimide‐functionalized HA, and maleimide‐PEG/furan‐functionalized HA have been employed to manufacture HA‐based hydrogels.^25^, ^64^, ^92^ To fasten the rate of Diels‐Alder reaction under physiological conditions, HA can be modified with the more electron‐rich methylfuran (Figure 8B,C).^88^ HA‐based hydrogels can be fabricated via inverse electron demand Diels‐Alder reaction as well under physiological conditions.^26^ In this reaction, tetrazine groups are used to react with other alkynes and alkenes. The hybrid molecule formulations can be injected and rapidly crosslinked in vivo because of the fast click reaction. For example, HA‐tetrazine and PEG‐norbornene were used to fabricate HA‐based hydrogels (Figure 8D). The crosslinking performance was highly influenced by temperature (Figure 8E).^89^ Besides, the Diels‐Alder reactions allow the combination of other crosslinking methods to produce dual‐crosslinked hydrogels. For instance, Wang and coworkers prepared a hydrogel of HA/PEG through thermal‐induced Diels‐Alder reaction and photo‐crosslinking. The mechanical property of this hydrogel gradually increased with the proceeding of the crosslinking process. The resultant hydrogel was used as cell carrier to promote cartilage repair since the loaded cells exhibited good viability in the experiment.^93^

#### 3.3.2 Schiff‐base reaction

**FIGURE 9:**
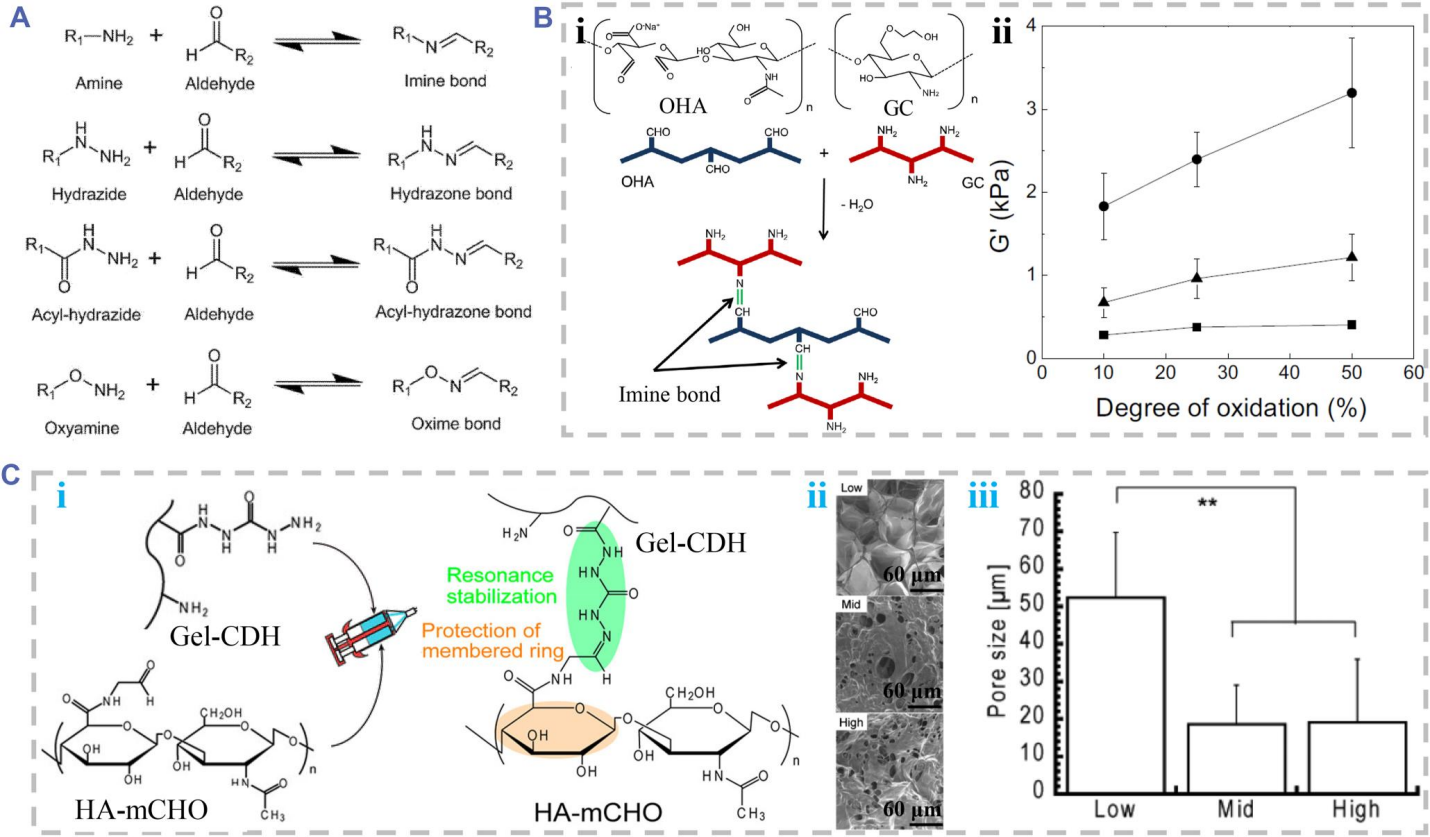
Schiff‐base reactions for dynamic covalent crosslinking. (A) Chemical reactions based on Schiff‐base reactions. Reproduced under terms of the CC‐BY license.^26^ Copyright 2021, The Authors, published by MDPI. (B) Fabrication of OHA/GC hydrogel. (i) The chemical structure of OHA and GC and the crosslinking diagram. (ii) The role of oxidation level of HA in the mechanical property of the resultant hydrogel. Reproduced with permission.^18^ Copyright 2017, Elsevier. (C) Fabrication of HA‐mCOH and Gel‐CDH. The principle diagram of HA‐mCOH and gelatin containing a hydrazide group. (i) The chemical structure and crosslinking diagram of HA‐mCOH and Gel‐CDH. (ii) SEM images of HA‐mCHO/Gel‐CDH hydrogels fabricated by different polymer concentrations. (iii) The pore size statistics of the HA‐mCHO/Gel‐CDH hydrogels fabricated by different polymer concentrations. Reproduced with permission.^94^ Copyright 2018, American Chemical Society.

The Schiff‐base reaction between nucleophilic amine groups and active carbonyl group can form oxime bonds, acylhydrazone bonds, hydrazone bonds, and imine bonds (Figure 9A). These dynamic covalent bonds can impart hydrogels with self‐healing properties and injectability for injection.^95^ Normally, the OHA was crosslinked into hydrogels through Schiff‐base reactions.^96^ The reported systems included OHA/cystamine dihydrochloride,^96^ OHA/CS,^97^ OHA/carboxyethyl‐chitosan (CEC),^98^ OHA/carboxymethyl chitosan,^99^ OHA/glycol chitosan (GC),^18^ CEC and OHA‐graft‐aniline tetramer polymer.^100^ For example, injectable hydrogels derived from GC and OHA were instantly formed in Dulbecco's phosphate‐buffered saline (Figure 9Bi). The mechanical properties of those hydrogels were changed by changing the oxidation degree of HA (Figure 9Bii).^18^ Besides, another strategy adopted active carbonyl groups to modify HA via pre‐modification, yielding a HA aldehyde (HA‐mCOH). The aldehyde group was used to react with hydrazide‐modified gelatin (Gel‐CDH) in phosphate‐buffered saline (Figure 9Ci). The porous structure and pore size of HA‐mCHO/Gel‐CDH hydrogels were determined by polymer concentrations (Figure 9Cii,iii).^94^ Similarly, the formation of oxime bonds through the reaction between oxyamine groups and aldehyde or ketone groups was also used to fabricate HA‐based hydrogels. As a typical case, Baker et al. developed the injectable hydrogels of HA aldehyde, HA ketone and oxyamine‐modified PEG. These hydrogels exhibited adjustable gelation times, controlled swelling ratio and great biocompatibility with retinal cells.^101^

With the advantages of needing no cross‐linking agents, ease of controlling the gelation process and mild reaction conditions, the Schiff‐base reaction is suitable to prepare injectable hydrogels.^29^ The resultant HA‐based hydrogels exhibit great potentials in biomedical applications due to their self‐healing capabilities and biocompatibility. However, they still face some drawbacks. For instance, imine chemistry‐induced hydrogel cannot serve as products requiring high gel stability for formed imine bonds can be easily hydrolyzed in acidic conditions.^29^ Besides, controlling the degradation rate of these hydrogels crosslinked by Schiff‐base reaction is still an uncontrollable issue in mass studies.^8^

#### 3.3.3 Thiol‐related reaction

**FIGURE 10:**
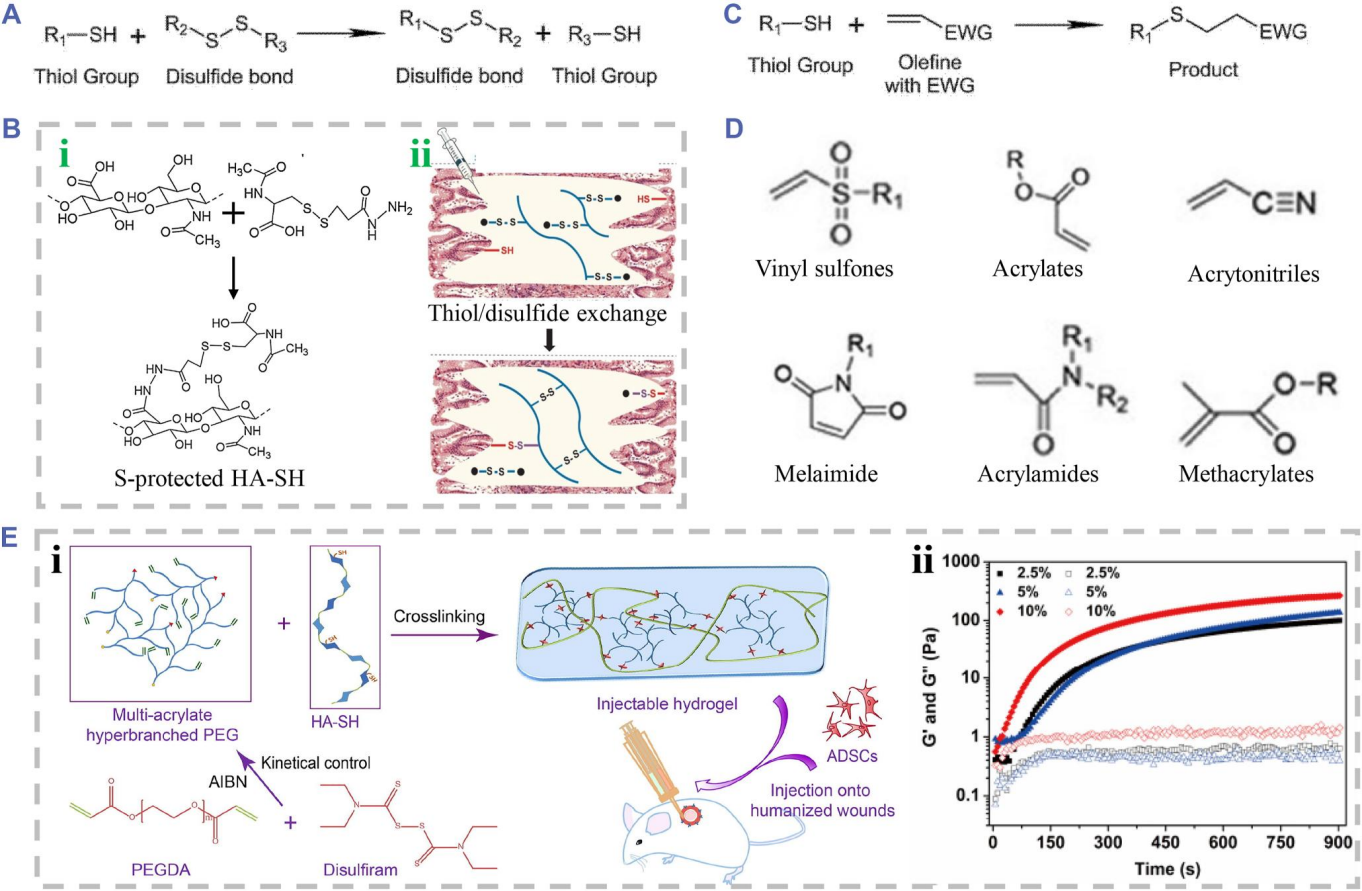
Thiol chemistry for covalent crosslinking. (A) Principle diagram of disulfide exchange reaction. Reproduced under terms of the CC‐BY license.^26^ Copyright 2021, The Authors, published by MDPI. (B) The fabrication of S‐protected HA‐SH based on disulfide exchange. (i) The synthesis principle and chemical structure of S‐protected HA‐SH. (ii) The principle diagram of crosslinking. Reproduced under terms of the CC‐BY license.^102^ Copyright 2020, The Authors, published by Elsevier. (C) Principle diagram of thiol‐Michael addition. Reproduced under terms of the CC‐BY license.^26^ Copyright 2021, The Authors, published by MDPI. (D) The useful vinyl groups used in reactions of thiol‐Michael addition. Reproduced under terms of the CC‐BY‐NC license.^6^ Copyright 2017, The Authors, published by SAGE Publications. (E) The fabrication of HP‐PEGs/HA‐SH hydrogel. (i) The principle diagram of crosslinking. (ii) Gelation process of HP‐PEGs/HA‐SH with different polymer concentrations. Reproduced with permission.^103^ Copyright 2018, Elsevier.

Parallel to disulfide formation of oxidation in Section 3.2.5, the free thiol groups can also undergo a reaction of disulfide exchange, endowing the hydrogels with self‐healing performance (Figure 10A).^26^ The HA‐based hydrogel crosslinked through disulfide exchange between HA‐SH and endogenous N‐acetyl cysteine or other thiol groups can be developed into cell culture scaffolds. Typically, Asim et al. synthesized S‐protected HA‐SH, which could be injected and crosslinked in vivo through disulfide exchange between polymers and thiol groups of endogenous molecules (Figure 10B).^102^ Besides, thiol groups were added to alkenes via Michael addition to fabricate HA‐based hydrogels under basic conditions, which was reversible (Figure 10C). When mixing vinyl‐ or thiol‐modified HA, the Michael addition reaction was proceeded through adjusting pH value to physiological level.^104^, ^105^ Polyethylene glycol diacrylate containing diacrylate group is traditionally utilized to crosslink HA‐SH by Michael addition reaction.^106^ Other useful vinyl groups involved in thiol‐Michael reactions were listed in Figure 10D.^6^ For instance, Xu et al. fabricated a novel injectable hydrogel of hyperbranched multi‐acrylated PEG (HP‐PEGs) and HA‐SH via thiol‐ene reaction under physiological conditions (Figure 10Ei). It exhibited tunable mechanical performance with the change of polymer concentration and reaction time (Figure 10Eii). It was used as a cell culture and delivery platform to encapsulate adipose‐derived stern cells (ADSCs), which could maintain ADSCs stemness and differentiation under the condition of induction media.^103^ Additionally, thiol‐Michael addition and photopolymerization can simultaneously exist in mild aqueous solution, which make it possible to design hydrogels with dual cross‐linkable capacities. When photopolymerization occurs in the alkaline solution and receives low light intensity, the reaction rates of thiol‐Michael addition and photopolymerization are comparable.^107^ Above hydrogels crosslinked by thiol‐related reactions present the potential to be developed as injectable materials, facilitating the applications for diverse biomedical purposes.^26^

#### 3.3.4 Boronic ester

**FIGURE 11:**
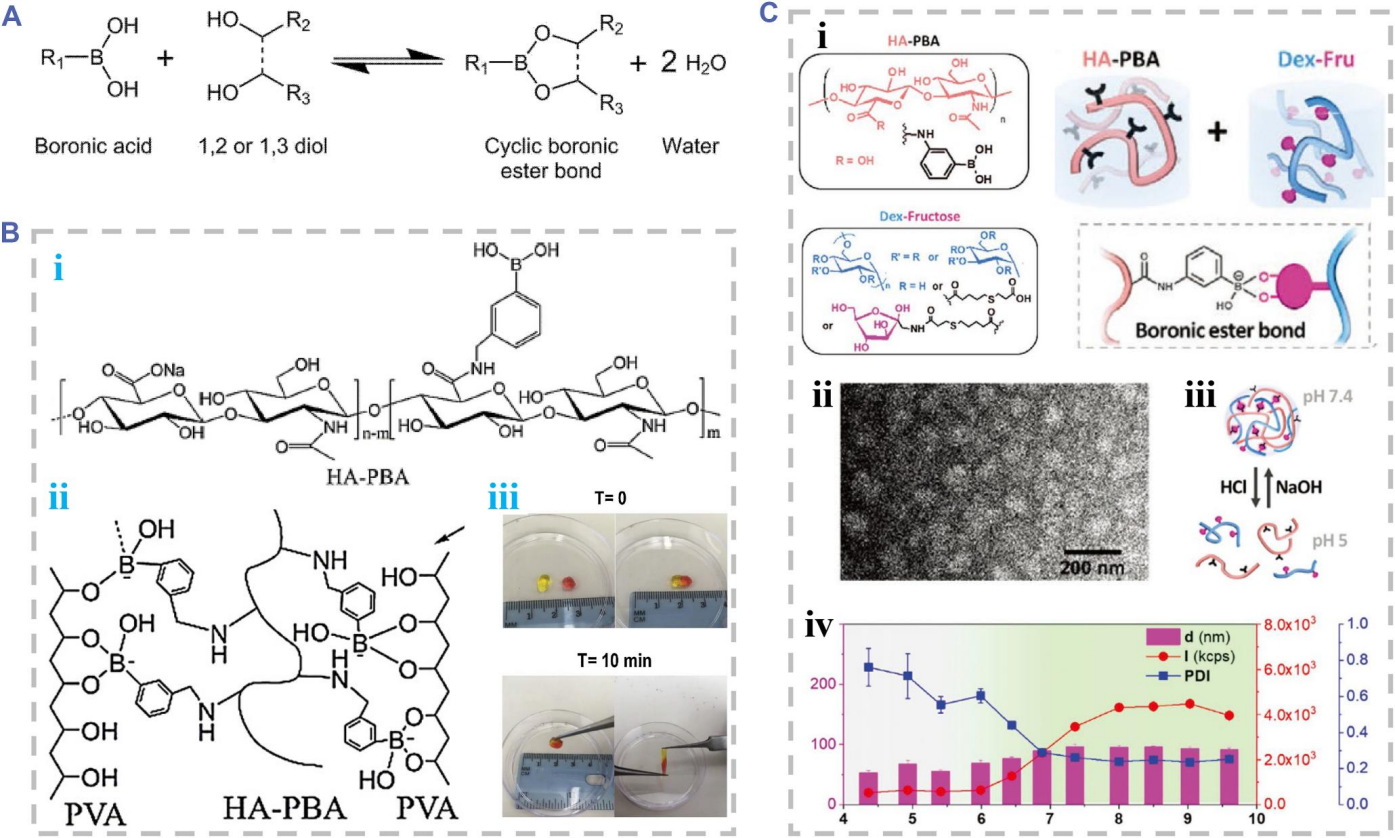
Boronic ester reactions for dynamic covalent crosslinking. (A) Principle diagram of the boronic ester reaction. Reproduced under terms of the CC‐BY license.^26^ Copyright 2021, The Authors, published by MDPI. (B) Self‐healing hydrogel of HA‐PBA/PVA. (i) The chemical structure of HA‐PBA. (ii) The chemical structure of HA‐PBA/PVA hydrogel. (iii) The optical images of hydrogel showing the self‐healing capacity. Reproduced with permission.^14^ Copyright 2020, Elsevier. (C) Nanogels of HA‐PBA/Dex‐Fru. (i) The chemical structure and crosslinking of HA‐PBA and Dex‐Fru. (ii) The TEM image of nanogels. (iii) Schematic diagram of the pH responsiveness of nanogels. (iv) Dynamic light scattering measurements of nanogels fabricated at different pH values. Reproduced with permission.^108^ Copyright 2020, John Wiley and Sons.

The condensation reactions between cis‐1, 3/cis‐1, 2 diols and boronic acids are efficient in an aqueous solution and under mild conditions, forming reversible boronic ester bonds (Figure 11A).^26^ The boronic esters are sensitive to pH value, which can experience hydrolysis in acidic condition. It was reported that phenylboronic acid (PBA, pKa ∼8.8)‐modified HA (HA‐PBA) and saccharide residues (e.g. maltose groups) modified HA can form dynamic hydrogels.^109^, ^110^ The crosslinking performance of HA‐PBA was related to the decrease in pKa of PBA by negatively charged carboxyl groups on HA chains. It is observed that the changes of the boronic acid species can contribute to changes of the reaction conditions, tailoring the hydrogel formation.^111^ Changing the substituent structure of the PBA, like introducing an EWG, may alter and decrease its pKa, allowing the reactions to occur at lower pH value (even physiological conditions). Based on the exchange of boronic acid/diol and boronate esters, the resultant hydrogels may perform the reversibility and self‐healing ability, which can be controlled by adding free glucose into the system and changing pH changes.^26^ When the pH value is close to the pKa value of boronic acids, this kind of reaction is most effective.

As typical cases, Shi and coworkers manufactured an injectable hydrogel of polyvinyl alcohol (PVA) and HA‐PBA, which was based on the dynamic boronic ester (Figure 11Bii). The gelation process cost less than 15 s at pH 7.4. This hydrogel possessed self‐healing capacities due to the dynamic bonds, which were suitable for injectability (Figure 11Biii). Based on the responsiveness of boronic ester to H_2_O_2_ at the biologically relevant concentration, this hydrogel could serve as a responsive drug delivery platform to H_2_O_2_.^14^ Velty et al. prepared nanogels through the reaction between HA‐PBA and fructose (Fru) or maltose moieties modified dextran (Dex) (Figure 11Ci,ii). The size of nanoparticles depended on the pH value of the reaction environment (Figure 11Ciii,iv).^108^

## 4 SUMMARY AND OUTLOOK

This review discussed the available modification strategies on HA and the versatile crosslinking strategies to create HA‐based hydrogels. The modifications mostly occur on reactive hydroxyl and carboxyl groups of HA, introducing new functional groups. The modified HA contributes to improving the physical/chemical properties of HA, which further facilitates the fabrication of HA‐based hydrogels with enhanced performance. Together with the advancement of various strategies including physical crosslinking, chemical crosslinking and DCC, a number of functional HA‐based hydrogels have been developed with more physiological functions and tailorable properties. These advantages have expanded their biomedical applications, ranging from advanced delivery systems for drug/cell delivery to tissue engineering scaffolds, etc.

However, there still exist several difficulties and challenges in the modification, fabrication and application of HA‐based hydrogels. Firstly, the exploration of HA modifications is insufficient. Several critical parameters such as precise selection of HA molecular weight, most effective substituted groups and optimal degree of substitution cannot be ignored and a comprehensive insight should be concentrated on these aspects. Besides, each crosslinked method has its limitations and a single crosslinking method still fails to satisfy the complex demands of biomedical applications. Advanced HA‐based hydrogels fabricated by multiple crosslinking are emerging to attract the attention of researchers due to their enhanced comprehensive performance. The issues are how to balance different reactions and precisely control the parameters, while ensuring a simple and easy synthesis system to generate safe products without toxic immunogenic properties. The last issue involves how to combine several fabrication methods and hydrogel formulations to obtain better comprehensive performance. Composite HA‐based hydrogels integrated with other functional components (e.g. organic components, inorganic materials, bioactive molecules and cells) need to be tailored to achieve superior performance. With the further efforts, it is believed that HA will have great possibilities to be transferred into commercial products, meeting the various market demands for therapeutics delivery, microbial reactors, scaffolds and detect device systems within the foreseeable future.

## AUTHOR CONTRIBUTIONS

Yunru Yu conceived the idea and revised the manuscript. Zhiqiang Luo conducted the investigation and wrote the manuscript. Yu Wang, Ye Xu and Jinglin Wang also revised the manuscript.

## CONFLICT OF INTEREST STATEMENT

The authors declare that there are no competing interests.
